# The AI Use in Healthcare Anxiety Scale (AI-HAS) for Adult Patients: Development, Validity and Reliability

**DOI:** 10.3390/healthcare14203520

**Published:** 2026-10-19

**Authors:** Özgür Selvi, Ali Yılmaz, Sakine Yalman, Özgen Korkmaz, Halis Altun

**Affiliations:** 1 Department of Audiovisual Techniques and Media Production, Kırıkkale Vocational School, Kırıkkale University, 71000 Kırıkkale, Türkiye; ozgurselvi@kku.edu.tr; 2 Department of Health Management, Faculty of Health Sciences, Kırıkkale University, 71000 Kırıkkale, Türkiye; 3 Department of Computer Technologies, Kırıkkale Vocational School, Kırıkkale University, 71000 Kırıkkale, Türkiye; sakineyalman@kku.edu.tr; 4 Department of Computer Engineering, Faculty of Engineering and Natural Science, Bandırma Onyedi Eylul University, 10000 Balıkesir, Türkiye; ozgenkorkmaz@bandirma.edu.tr; 5 Department of Software Engineering, Faculty of Engineering and Natural Science, Istanbul Health and Technology University, 34000 İstanbul, Türkiye; halis.altun@istun.edu.tr

**Keywords:** healthcare, artificial intelligence, anxiety, adult patients

## Abstract

**Background/Objectives:** Artificial intelligence (AI) is increasingly used in healthcare, but concerns about diagnostic errors, data security, reduced human interaction, and opaque algorithmic decisions may cause patient anxiety and hinder acceptance. This study developed and psychometrically validated the Artificial Intelligence Use in Healthcare Anxiety Scale (AI-HAS) for adult outpatients. **Methods:** Following DeVellis’s eight-step framework, a three-member expert panel comprising two psychologists with PhDs in psychology and an expert in AI applications in education with a PhD in educational technology generated 35 items. Cognitive debriefing and two rounds of formal expert review reduced the pool to 25 items. Participants were recruited by convenience sampling at Kırıkkale University Faculty of Medicine Hospital, Türkiye. Exploratory factor analysis included 369 participants after excluding 13 multivariate outliers identified using Mahalanobis distance (*p* < 0.001). Confirmatory factor analysis used an independent sample of 225 participants. Test–retest reliability was assessed in 26 participants after approximately 30 days using Pearson’s correlation. **Results:** Exploratory factor analysis supported an 18-item, four-factor structure explaining 65.80% of the variance: Anxiety Regarding Negative Outcomes, Security Anxiety, AI Integration Anxiety, and Accuracy Anxiety. Confirmatory factor analysis yielded χ^2^(df, *p*) = 328.4 (128), *p* < 0.001; RMSEA = 0.082 [0.074, 0.097]; CFI = 0.930; TLI = 0.916; and SRMR = 0.056. Cronbach’s alpha and McDonald’s omega coefficients ranged from 0.83 to 0.91. **Conclusions:** The AI-HAS showed preliminary evidence of validity and reliability for measuring adult outpatients’ anxiety about AI use in healthcare. Further validation in more representative samples is warranted.

## 1. Introduction

Artificial Intelligence (AI) is increasingly used in healthcare to analyze medical data and support clinical and administrative tasks [1,2]. Applications include medical image analysis, patient monitoring, electronic health record management, and clinical decision support [1,2]. The use of AI in healthcare raises concerns about algorithmic bias, privacy, data security and accountability for errors [3,4]. Patients also express concerns about diagnostic safety, autonomy, healthcare costs and the protection of their personal information [5]. These concerns provide a context for examining patients’ anxiety about AI-supported diagnosis, treatment decisions, and other healthcare services, including the reliability of AI systems and the extent of human oversight [5,6].

Anxiety involves worry, tension and apprehension in response to perceived threats and persistent anxiety can affect functioning and quality of life [7,8]. The Generalized Anxiety Disorder-7 (GAD-7) serves as a brief, psychometrically validated self-report screening tool designed to monitor anxiety severity [8]. Similarly, the State-Trait Anxiety Inventory (STAI) assesses state and trait anxiety, while the Hospital Anxiety and Depression Scale (HADS) assesses symptoms of anxiety and depression [9]. These instruments provide measures of general anxiety symptoms or dispositions, but their content does not specifically address patients’ concerns about AI use in their healthcare. The construct examined here concerns anxiety directed toward a particular technology and context. It does not imply a clinical anxiety disorder.

In healthcare, AI-related anxiety may involve worry and apprehension about the use of AI technologies and their potential adverse consequences [5]. In this study, AI use anxiety in healthcare is defined as adult patients’ worry and apprehension about the use of AI systems in diagnosis, treatment decisions, and other aspects of their care. AI distrust concerns negative expectations about an AI system’s reliability or behavior [10], whereas algorithm aversion concerns reluctance to rely on algorithmic recommendations [11]. Health anxiety concerns worry about illness [12], while cyberchondria involves excessive online health-information seeking associated with distress [13]. These constructs may overlap but are not interchangeable. AI use anxiety in healthcare is conceptualized here as a context-specific expression of broader AI anxiety, without assuming empirical independence from related constructs.

Patient studies provide evidence about acceptance and preferences concerning clinical AI. Robertson et al. [14] found that information about diagnostic accuracy, physician recommendations, and opportunities to be heard influenced patients’ choices between AI and human providers. Palmisciano et al. [15] reported acceptance of several AI applications in neurosurgery alongside reservations about autonomous surgery and a preference for continued surgeon control. These findings identify relevant concerns but should not be interpreted as direct evidence of anxiety severity. Neither study developed an AI anxiety scale specifically for adult patients in healthcare settings. AI anxiety has also been investigated among healthcare professionals [16]. However, findings from professional populations do not establish how AI use anxiety should be measured among adult patients receiving care.

Validated instruments exist for AI-related anxiety and fear [17,18]. Wang and Wang (2022) developed the Artificial Intelligence Anxiety Scale that includes learning, job replacement, sociotechnical blindness, and AI configuration dimensions [17]. Corradi et al. (2026) developed the Fears Towards Generative Artificial Intelligence Scale to assess fears concerning generative AI, including its societal implications [18]. These instruments provide relevant comparisons, but their original validation studies do not establish their suitability for assessing adult patients’ anxiety about AI use in their own healthcare [17,18]. The measurement need addressed here concerns the fit between the construct, item content, and patient population, including the representation of clinical safety, confidentiality, individualized care, and human involvement [5,6,19]. A focused assessment of anxiety in this patient population can therefore complement existing measures developed for broader uses of AI.

The cognitive appraisal perspective provides an interpretive framework for these concerns by distinguishing appraisals of what is at stake from appraisals of available coping options [20]. Applied conceptually to healthcare AI, this perspective suggests that anticipated harm to health, confidentiality, or autonomy may become a source of anxiety when patients perceive limited opportunities to understand, question, or influence its use. This interpretation connects the perceived consequences of AI use with the availability of explanation, professional support, and human oversight; these relationships are theoretical propositions rather than causal effects tested in the present study.

This perspective and the patient-concern literature highlight several potentially overlapping content areas relevant to the proposed construct. Anticipated negative consequences include concerns about unfair or insufficiently individualized care and the displacement of human roles in healthcare [5,17]. Safety and security concerns include both the misuse of personal information and the risk of incorrect clinical decisions [5,6]. Concerns about mechanization relate to diminished human involvement, communication, and oversight [14,15]. A further content area concerns patients’ apprehension about the accuracy and presentation of AI-supported clinical information, particularly when diagnostic recommendations are expressed with unwarranted certainty [21,22]. In the present study, this also encompasses concern that apparently definitive health information could intensify preoccupation with health. These themes clarify the proposed content of the construct without imposing a predetermined factor structure; the number and composition of dimensions were investigated empirically, consistent with an exploratory approach to scale development [19].

The present study aims to develop the Artificial Intelligence Use in Healthcare Anxiety Scale (AI-HAS) for adult outpatients and examine its dimensional structure, internal consistency, and test–retest reliability. Its intended contribution is initial psychometric evidence for a patient-focused measure, with interpretation grounded in the content of its items and the population in which it was evaluated.

## 2. Method

### 2.1. Research Design

This study utilizes a psychometric scale development design aimed at quantitatively evaluating adult outpatients’ anxiety regarding the integration and deployment of artificial intelligence within healthcare services. To achieve this objective, a structurally grounded theoretical framework was established, and the scale refinement process was iteratively driven by empirical data derived from two distinct participant cohorts allocated respectively to Exploratory Factor Analysis (EFA) and Confirmatory Factor Analysis (CFA) [23]. This methodological configuration was specifically engineered to address the empirical deficit for a psychometrically robust, multidimensional, and standardized measurement instrument capable of mapping AI-induced anxiety in patient populations.

### 2.2. Study Group

The target population for this investigation comprises adult outpatients admitted to healthcare facilities in Türkiye. The heterogeneous and expansive nature of this population serves as a critical asset, fundamentally enhancing the external validity, generalizability, and psychometric stability of the developed instrument [19]. To ensure structural independence across analyses, distinct non-overlapping samples were drawn for the EFA and CFA phases. For the temporal stability (test–retest) analysis, a longitudinal subsample was established by securing the electronic contact information of individuals who had previously completed the EFA data collection phase; follow-up assessments were subsequently initiated after an approximate 30-day temporal interval. Participants across all cohorts were selected via a non-probability convenience sampling strategy based on voluntary participation.

- **EFA Sample:** The calibration cohort for the EFA comprised *n* = 382 adult outpatients who were admitted to various outpatient clinics at the Kırıkkale University Faculty of Medicine Hospital in Türkiye between 1 March and 31 March 2026, and who provided formal informed consent. Prior to executing the factor analytic procedures, the dataset was rigorously screened for statistical anomalies. Following multivariate outlier detection using Mahalanobis distance (Mahalanobis distance: Observations exceeding the critical chi-square value at the *p* < 0.001 significance level were considered multivariate outliers and removed from the dataset), 13 cases were identified as aberrant and excluded, yielding a final analytical sample of *n* = 369.
- **CFA Sample:** The validation cohort for the CFA consisted of *n* = 225 adult outpatients who presented to diverse outpatient clinics at the Kırıkkale University Faculty of Medicine Hospital in Türkiye between 1 April and 1 May 2026, and voluntarily enrolled in the study. To preserve the mathematical independence of the confirmatory model, data were strictly harvested from outpatients who had no prior exposure to the earlier phases of this research.
- **Stability Analysis Sample:** The longitudinal sample for assessing test–retest reliability initially targeted 30 adult outpatients from the EFA pool who had consented to follow-up contacts. The secondary evaluation was administered electronically via email between 1 May and 15 May 2026. A total of *n* = 26 patients completed and returned the follow-up instrument, representing an acceptable response retention rate for temporal stability tracking. Detailed demographic distributions across these three independent analytical cohorts are presented in Table 1.

As illustrated in Table 1, empirical data were distinctively collected from 369, 225, and 26 outpatients across the three separate analytical phases, ensuring localized demographic variation across all validation tiers. Thus, demographic diversity at the local level has been ensured across all verification stages. Regarding the data collected, 50.4% for EFA, 49.7% for CFA, and 61.5% for stability analyses were obtained from women, while the remainder came from men. Furthermore, 78.6% of the data for EFA, 80.4% for CFA, and 76.9% for stability analyses were gathered from outpatients residing in the city center. In terms of education level, the largest groups among the participating outpatients across all three stages consisted of individuals holding a bachelor’s degree and high school graduates, respectively. Regarding age distribution, outpatients in the 20–30 age range constituted the largest group across all three stages, whereas patients in the 18–19 age group made up the smallest group.

### 2.3. Scale Development Process

The systematic construction of the measurement instrument strictly adhered to the eight-step scale development framework operationalized by DeVellis [24]. The structural execution of each sequential phase is detailed below:

1. Construct Operationalization: This study conceptualizes the anxiety experienced by adult outpatients regarding AI applications in healthcare as a multidimensional construct. To establish initial semantic boundaries, the foundational theoretical underpinnings of affective and technological anxiety were thoroughly reviewed. The scope was then contextualized around how outpatients experience psychological distress when automated systems interface with their clinical diagnoses and therapies. This conceptual perimeter is detailed in the introductory section. Crucially, to prevent premature structural restrictions and maximize the comprehensiveness of the item pool, potential latent factors were not a priori imposed; instead, the underlying dimensions were permitted to emerge organically from subsequent empirical factor extractions.
2. Item Generation Pool: The latent indicator matrix was developed by a multidisciplinary research panel comprising five academic experts across three universities (including three Full Professors, one Associate Professor, and one Assistant Professor) specializing in educational technology, computer engineering (*n* = 2), health communication, and healthcare management. Adhering to the psychometric principle of over-inclusive item generation to guarantee adequate structural coverage [24], the panel generated an initial item pool of 35 indicators, adhering to psychometric conventions of over-inclusive item generation to guarantee comprehensive domain sampling. Items were meticulously designed to meet precise criteria: sentences were constructed to be clear, unifocal, grammatically straightforward, and anchored within daily conversational idioms that represent apparent clinical awareness. Ambiguous syntax, negative or double-negative inflections, and multi-barreled assertions were strictly avoided [25]. This rigorous optimization for content and face validity aimed to maximize the cognitive, affective, and behavioral integrity of the measurement matrix [26].
3. Response Format Selection and Target Audience Debriefing: During this stage, the measurement parameter for capturing participant responses was defined. Given its direct implications for structural validity and reliability estimation [24], a continuous-interval analog response format was deemed optimal, leading to the selection of a traditional 5-point Likert scale. This configuration provides sufficient mathematical granularity for adult cohorts while preserving meaningful semantic discrimination between response thresholds [19]. The response options were anchored as follows: *1* *= Strongly Disagree, 2 = Disagree, 3 = Moderately Agree, 4 = Agree, 5 = Strongly Agree*. This symmetrical gradation is highly effective for mitigating systematic response bias, such as central tendency and acquiescence effects [25]. Following format assembly, a pilot cognitive debriefing was conducted with seven undergraduate students enrolled in a Faculty of Health Sciences, moderated directly by two principal investigators. All of these students are fourth-year nursing students who have completed the Public Health Nursing course. Although this group of students does not fall directly within the target audience, it was considered that—given their foundational skills in public health—they were well-equipped to provide adequate feedback on the clarity of the items. Therefore, these students were selected. During these 15-min diagnostic sessions, students were instructed to read every item carefully and flag expressions that introduced semantic ambiguity, unfamiliar jargon, or syntactical fragmentation. The feedback logs and debriefing notes were scrutinized by the research team during a subsequent synchronous session. Based on this qualitative analysis, adjustments were made to the linguistic clarity and face validity of the scale, resulting in the elimination of two items that failed to satisfy the requisite psychometric criteria.
4. Expert Panel Evaluation and Scale Refinement: To establish robust content validity and ensure strict alignment with the latent theoretical construct, the refined item matrix was subjected to formal expert review. The pool of 33 draft items was independently evaluated by an external panel consisting of two expert psychologists holding PhDs in psychology and an expert in AI applications in education holding a PhD in educational technology. Experts were asked to base their evaluations of the items on criteria such as item overlap, the extent to which an item expresses anxiety, and content validity. Panelists quantified each item using a trichotomous diagnostic index (*Appropriate, Requires Revision, Inappropriate*) and provided qualitative enhancement critiques where necessary. For six of these items, all panelists expressed the view that they should be revised; for two items, one panelist deemed them appropriate, while two panelists recommended revision. The research team reviewed these results and decided to revise all eight items. Following the initial review cycle, the research team implemented targeted linguistic and conceptual revisions across eight items. It was recommended that five items—for which panelists identified significant conceptual overlap (with all experts advising removal for three items, and two experts advising removal while one advised revision for the other two)—and three additional items—for which all experts advised removal due to a lack of clear indicators of anxiety or weak thematic alignment—be excluded from the scale. The research team reviewed the expert opinions and, concluding that the removal of these items would not compromise content validity, decided to exclude all eight items from the scale. The modified 25-item matrix was re-evaluated by the same panel, yielding absolute consensus regarding its theoretical suitability. Finally, to ensure impeccable grammatical compliance, the draft was scrutinized by a scholar of Turkish Linguistics, resulting in minor word substitutions across two items to optimize semantic precision.
5. Pilot Administration and Exploratory Factor Analysis (EFA): The finalized 25-item draft was administered to the calibration sample of 369 outpatients across various clinical departments. As emphasized by Tabachnick and Fidell [27], evaluating sample adequacy requires a concurrent assessment of the item-to-respondent ratio alongside the overall quality of the data matrix. The primary objective of this phase was to subject the empirical data to rigorous psychometric screening, thereby establishing substantial structural validity evidence and isolating a refined latent factor matrix [28]. The calibration sample must maintain sufficient statistical power and representativeness to accurately uncover the true latent construct and minimize standard errors of measurement [29]. Consistent with the widely accepted psychometric convention recommending a minimum sample size of 10 respondents per item [28], our sample of 369 for a 25-item draft comfortably exceeded this criterion, ensuring a robust foundation for structure validation and high internal consistency. Additionally, selecting adult outpatients directly experiencing healthcare environments ensured that the sample accurately reflected authentic user awareness.

To uncover the latent structure, Principal Component Analysis (PCA) was performed. While PCA is widely used for dimension reduction, it served here to establish the exact number of retainable components. In the factor rotation stage, the oblique rotation method was first tested to observe possible relationships between factors, and the correlations between factors are presented in Table 2.

As shown in Table 2, with the exception of the correlation between the second and fourth factors (a value that is also very close to the critical threshold), the correlations among the other factors fell below the critical threshold (r < 0.32) [27]. Therefore, the Varimax orthogonal rotation technique, which yields more stable and interpretable factor loadings, was preferred.

The extraction criteria were evaluated using a comprehensive suite of psychometric indicators, including the Kaiser–Meyer–Olkin (KMO) measure of sampling adequacy, Bartlett’s test of sphericity, corrected item-total correlations, structural factor loadings, scree plots, and total explained variance. This process aimed to identify and eliminate items displaying poor primary factor loadings (less than 0.40) or cross-loading anomalies across multiple dimensions. Removing these psychometrically deficient indicators structurally strengthens the scale, ensures a parsimonious configuration, and enhances conceptual interpretability [30]. Crucially, statistical criteria were balanced with theoretical alignment to ensure that the emerging dimensions remained conceptually coherent. Following structural refinement, the scale’s internal consistency was evaluated by calculating both Cronbach’s alpha and McDonald’s omega coefficients for each isolated factor and the overall instrument. While Cronbach’s alpha provides a classic assessment of inter-item homogeneity under tau-equivalence assumptions, McDonald’s omega delivers a more accurate, robust reliability estimate by accommodating varying factor loadings. Utilizing both coefficients provides comprehensive, rigorous evidence regarding the internal scale consistency [31].

6. Final Scale Construction: Following the EFA and the systematic application of the elimination criteria (detailed in the results section), the instrument was refined to an 18-item, four-factor structural solution. The research team carefully reviewed this four-dimensional configuration against the theoretical framework, confirming total conceptual harmony. The latent factors were subsequently named based on the underlying themes of their respective items.
7. Confirmatory Factor Analysis (CFA): To evaluate the structural validity, generalizability, and replicability of the four-factor model derived from the EFA, an independent validation sample of 225 adult outpatients was gathered. The sample size comfortably exceeded the psychometric threshold of 10 participants per item for an 18-item scale, providing adequate statistical power to test the hypothesized covariance structure [27]. The CFA evaluated standardized factor loadings, error variances, and global model fit indices to determine whether the empirical data fit the hypothesized theoretical framework [32]. As noted by Brown [33], confirming an EFA-derived structure using an independent CFA sample provides exceptionally strong evidence for construct validity. The analysis demonstrated an excellent structural fit, with the items loading onto their designated factors with high statistical precision and strong parameter estimates.
8. Temporal Stability Assessment: While the aforementioned procedures provide comprehensive evidence of internal consistency and construct validity, they do not establish the temporal stability of the scores over time [34]. To address this, the scale was re-administered to a longitudinal sub-cohort of 26 adult outpatients approximately one month after the primary validation phase. The temporal stability coefficient was calculated using the Pearson r correlation coefficient between the test and retest scores, testing the null hypothesis that the instrument’s scores fluctuate due to random error over time.

### 2.4. Ethical Considerations

Administrative and ethical clearance for this study was officially granted by the Kırıkkale University Social and Human Sciences Scientific Research and Publication Ethics Committee during Session 01 on 20 January 2026 (Decision No: 20.01.2026-01), with formal authorization communicated via official directive No. 407583 on 25 February 2026. Additionally, formal institutional permission to conduct the study within the clinical environment was secured from the Chief Physician’s Office of the Kırıkkale University Faculty of Medicine Hospital on 26 February 2026 (Document No: E.408648). Prior to data collection, all adult outpatients were thoroughly briefed on the research scope, objectives, and data usage protocols. Participation was entirely voluntary, and only individuals who provided formal informed consent were enrolled. The primary data collection instruments contained no personally identifiable information (PII), guaranteeing absolute participant anonymity throughout the analytical process. Furthermore, due to the requirements of the test–retest methodology, electronic mail addresses were collected from a limited subsample. To safeguard participant privacy, this contact information was permanently purged from all storage repositories immediately upon the completion of the longitudinal data collection phase, ensuring no external exposure or data sharing occurred.

## 3. Findings

### 3.1. Psychometric Validation Evidence

To evaluate the psychometric properties of the Artificial Intelligence Use in Healthcare Anxiety Scale (AI-HAS) for adult outpatients, construct validity, corrected item-total correlations, and item discrimination indices were systematically scrutinized. The empirical findings are detailed below.

### 3.2. Construct Validity

#### 3.2.1. Exploratory Factor Analysis (EFA) as Principal Component Analyses

Prior to assessing the latent structure of the AI-HAS, the primary data matrix was tested for factorability using the Kaiser–Meyer–Olkin (KMO) measure of sampling adequacy and Bartlett’s test of sphericity. The initial analysis yielded a KMO index of 0.929 and a highly significant Bartlett’s test (*χ*^2^ = 5893.952; *df* = 300; *p* < 0.001), confirming that the correlation matrix possessed sufficient shared variance to warrant a rigorous principal component framework on the initial 25-item pool.

In the primary phase, a Principal Component Analysis (PCA) was executed to explore the baseline dimensionality of the instrument. Factor extraction was guided by Varimax orthogonal rotation. While the initial solution revealed no items with primary factor loadings below the baseline threshold of 0.40, five distinct latent dimensions emerged with eigenvalues greater than 1.0. However, to achieve greater parsimony, the scree plot trajectories and cumulative variance contributions were carefully scrutinized, suggesting that a four-factor solution provided a more theoretically coherent structure.

During this refinement, seven items exhibited prominent cross-loading profiles (fluid variances distributed across multiple dimensions). To protect content validity, these items were subjected to close semantic review. The research team consulted an educational measurement and evaluation expert alongside a public health specialist to determine if removing or reallocating these indicators would compromise the scale’s target domain. The panel confirmed that the essential content remained secure. Following iterative analytical eliminations, a stable four-factor configuration was established, where the dimensions contained seven, four, four, and three items respectively.

Without rotation, the unrotated factor loadings for the final 18 items ranged between 0.427 and 0.779. Following the application of the Varimax orthogonal rotation, the rotated factor loadings improved significantly, ranging from 0.506 to 0.812. A secondary factorability check on this refined 18-item structure confirmed excellent sampling adequacy (KMO = 0.903) and strong matrix sphericity (*χ*^2^ = 3648.652, *df* = 153, *p* < 0.001). Crucially, this 18-item, four-factor framework accounted for 65.80% of the total explained variance, surpassing standard psychometric recommendations for behavioral constructs.

This dimensional distribution was further supported by the scree plot, which illustrates the eigenvalue drop-off for each extracted component.

**Figure 1:**
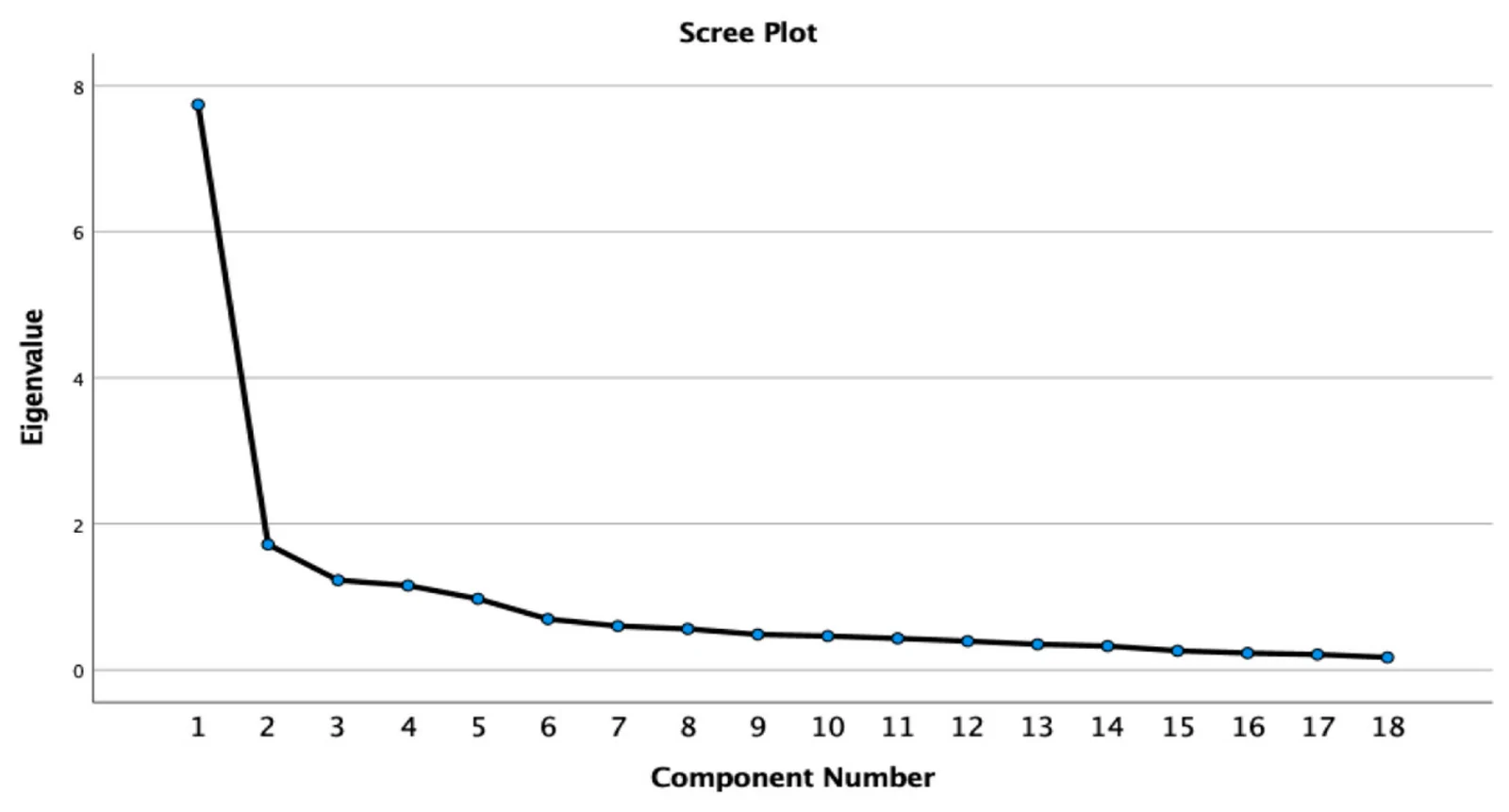
Scree Plot.

As shown in Figure 1, a sharp downward inflection occurs through the first four factors, highlighting their substantial contribution to the variance. Beyond the fourth component, the curve flattens into a linear plateau, meaning that subsequent factors contribute negligible, nearly identical increments to the explained variance [35,36]. The final factor loading matrix, item-level commonalities, component eigenvalues, and dimension-specific variance proportions are detailed in Table 3.

The semantic boundaries of the four emerging factors were conceptualized by evaluating the core themes of their constituent items. Factor 1 isolates patient concerns regarding systemic disruption, over-reliance, and depersonalization, and was named *Anxiety Regarding Negative Outcomes*. Factor 2 covers items linked directly to data vulnerability, clinical error, and digital malfeasance, and was designated *Security Anxiety*. Factor 3 addresses the loss of human agency and the erosion of the therapeutic alliance and was defined as *AI Integration Anxiety*. Factor 4 captures patient distress regarding the rigid or absolute presentation of health data and was termed *Accuracy Anxiety*.

The initial factor captured the largest portion of variance with an eigenvalue of 3.38 (explaining 18.80% of the variance). The second factor had an eigenvalue of 3.13 (explaining 17.37%), followed by the third factor with an eigenvalue of 2.71 (explaining 15.05%), and the final factor with an eigenvalue of 2.62 (explaining 14.58%). Together, these dimensions provide a structurally comprehensive framework for measuring healthcare AI anxiety.

#### 3.2.2. Confirmatory Factor Analysis (CFA)

To evaluate the structural validity, replicability, and parameter stability of the EFA-derived model, a Confirmatory Factor Analysis (CFA) was conducted using an independent validation sample (*n* = 225). The Maximum Likelihood (ML) method was employed for the Confirmatory Factor Analysis (CFA). The standard ML method used by AMOS assumes that the data are continuous and follow a multivariate normal distribution. Accordingly, this assumption was tested for each item in the scale by examining the kurtosis and skewness coefficients; it was observed that skewness coefficients ranged from −0.745 to 0.255, while kurtosis coefficients ranged from −1.252 to −0.503. Based on these findings, it was determined that the data for each item in the scale were normally distributed. Global model fit statistics were computed to evaluate the model’s congruence with the empirical covariance matrix, as summarized in Table 4.

The structural model demonstrated favorable fit metrics, characterized by a CMIN/DF ratio of 2.510 and a Root Mean Square Error of Approximation (RMSEA) of 0.082. These results suggest an acceptable model fit. Similarly, the comparative fit index (CFI = 0.930), incremental fit index (IFI = 0.931), and Tucker–Lewis index (TLI = 0.916) consistently met established psychometric thresholds, confirming that the hypothesized measurement model aligns well with the verification data [27,37]. In short, the CFA confirms the structural stability of the four-factor instrument. The standardized regression weights and parameter estimates computed for each indicator are detailed in Table 5.

As presented in Table 5, all standardized parameters demonstrated strong statistical associations with their target dimensions. The latent indicators loaded heavily onto their respective factors, with standardized regression weights ranging from 0.572 to 0.758 for *Anxiety Regarding Negative Outcomes*, 0.662 to 0.827 for *Security Anxiety*, 0.776 to 0.910 for *AI Integration Anxiety*, and 0.713 to 0.891 for *Accuracy Anxiety*. The results regarding the convergent validity and composite reliability of the scale dimensions are summarized in Table 6.

Composite Reliability (CR) and Average Variance Extracted (AVE) values were examined to determine the convergent validity of the four-factor measurement model (Table 5). The analysis results indicated that the CR values for the sub-dimensions ranged from 0.852 to 0.916, all exceeding the critical threshold of 0.70. An examination of the AVE values revealed that the dimensions F2 (0.596), F3 (0.733), and F4 (0.689) scored above the acceptable lower limit of 0.50. Although the AVE value for the F1 dimension (Anxiety Regarding Neg. Outcomes) was borderline (0.450), convergent validity was deemed established because the CR value for this dimension (0.852) was well above the target threshold. These findings statistically support the construct validity and internal consistency of the sub-dimensions of the developed scale.

#### 3.2.3. Item-Factor Correlations

To evaluate how effectively each latent indicator captures the overarching construct, item-factor correlations were calculated. This procedure assesses the alignment of individual items with their assigned subscales. The resulting coefficients are presented in Table 7.

**Figure 2:**
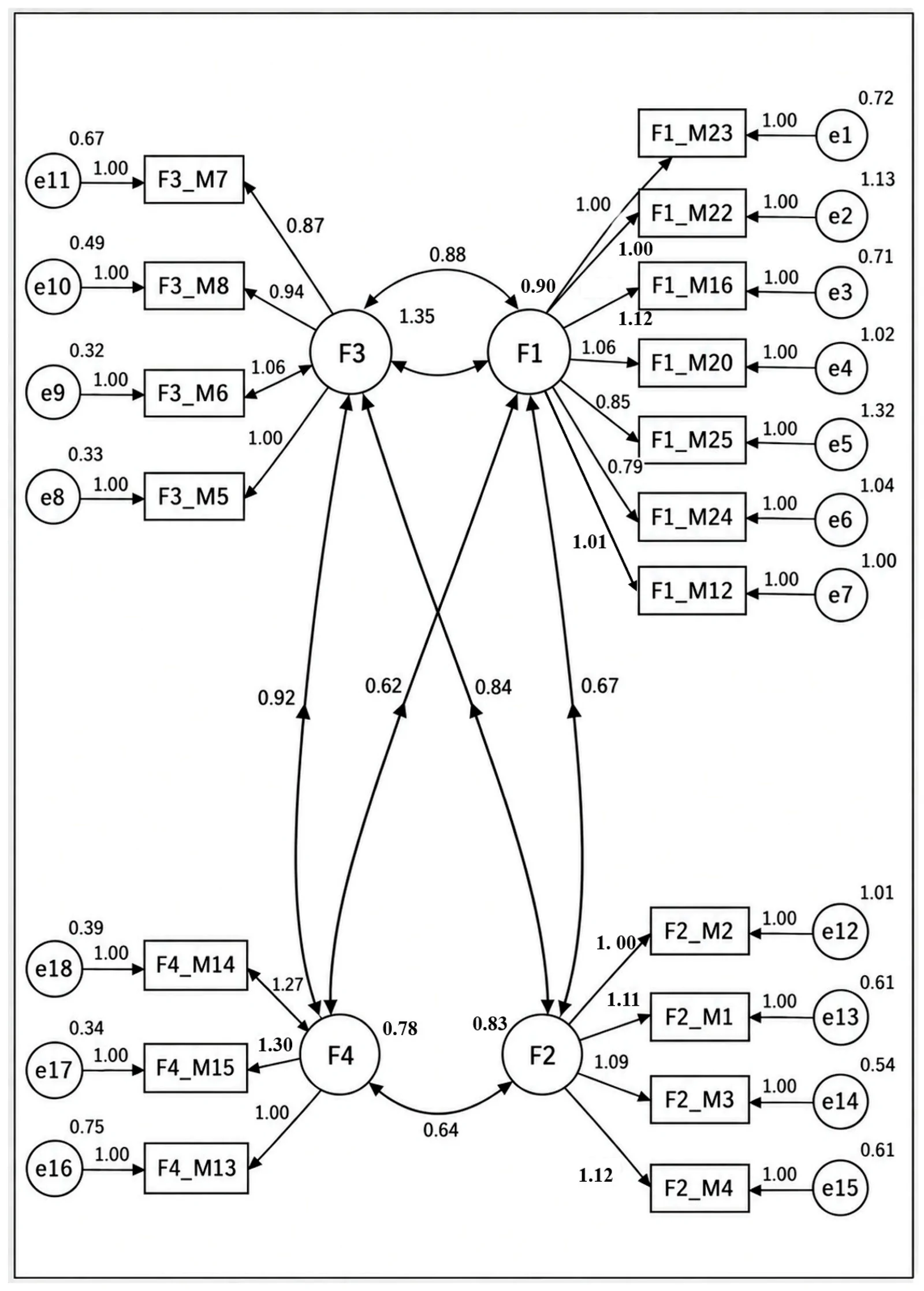
Structural Equation Model Diagram for the Confirmatory Factor Analysis.

As shown in Table 7, the item-to-subscale correlation coefficients ranged from 0.561 to 0.785 for Factor 1, 0.811 to 0.870 for Factor 2, 0.805 to 0.870 for Factor 3, and 0.819 to 0.900 for Factor 4. Every item demonstrated a strong, positive, and statistically significant association with its target subscale (*p* < 0.001), confirming that each item contributes effectively to the overall purpose of the scale. Figure 2 illustrates the structural equation model used in the confirmatory factor analysis (CFA).

#### 3.2.4. Item Discrimination Power

To determine whether the items could effectively differentiate between individuals experiencing high versus low levels of healthcare AI anxiety, an item discrimination analysis was performed. The total raw scores were sorted, and upper and lower 27% groups (*n* = 100 per group) were established. Independent-samples t-tests were then calculated to compare item means between these extreme groups, as summarized in Table 8.

The independent-samples t-test values for individual items, subscales, and the overall scale score ranged from 7.59 to 22.70. The overall instrument demonstrated a high discrimination index (*t* = 30.47), with every mean difference reaching statistical significance (*p* < 0.001). These results show that both the overall scale and its individual items possess excellent discriminative power.

### 3.3. Reliability Analysis

#### Internal Consistency and Temporal Stability

To evaluate the reliability of the scale, internal consistency was assessed using Cronbach’s alpha and McDonald’s omega coefficients, while temporal stability was estimated via test–retest correlation. The resulting reliability metrics are detailed in Table 9.

The overall 18-item scale demonstrated high internal consistency, with a Cronbach’s alpha of 0.918, a McDonald’s omega of 0.916, and an overall stability coefficient (Pearson r) t of 0.409. For the subscales, Cronbach’s alpha values were 0.836, 0.864, 0.847, and 0.837 respectively, while McDonald’s omega values were 0.837, 0.853, 0.843 and 0.846, indicating robust internal homogeneity across all subscales.

The test–retest reliability coefficients across a 30-day interval ranged from 0.389 to 0.458. While these internal consistency metrics demonstrate excellent reliability, the lower longitudinal stability suggests that healthcare AI anxiety may operate partly as a state-like construct. Rather than functioning purely as a stable, time-independent trait, this type of anxiety can fluctuate based on current media reporting, evolving public discussions, or a patient’s immediate health status during assessment, which may have changed their anxiety levels after 30 days. Additionally, the small size of the follow-up sample and the shift to email data collection (away from the clinical environment) may have introduced minor measurement variance. Taking these factors into account, the findings show that the scale provides highly consistent internal measurements along with acceptable temporal stability, despite the slightly lower correlation scores.

## 4. Discussion

The rapid assimilation of artificial intelligence (AI) into clinical workflows has underscored the critical urgency for developing robust, psychometrically sound instruments to evaluate patient-side perceptions, particularly anxiety. This study developed and validated the “Artificial Intelligence Use in Healthcare Anxiety Scale” (AI-HAS), resulting in an 18-item, four-factor instrument purposely engineered for adult outpatient populations. The exploratory factor analysis (EFA) and subsequent confirmatory factor analysis (CFA) demonstrated that the AI-HAS possesses robust structural validity and high internal consistency, successfully filling a prominent gap in the contemporary medical psychometrics and health informatics literature.

The first and third dimensions, *Anxiety Regarding Negative Outcomes* and *AI Integration Anxiety*, collectively reflect deep-seated concerns regarding the erosion of the “human touch” in medicine. In the medical sociology and communication literature, the patient-physician relationship is conceptualized as a cornerstone of therapeutic efficacy [5]. The emergence of *AI Integration Anxiety* highlights patients’ dread of clinical depersonalization, where their unique, idiosyncratic clinical narratives might be reduced to binary processing by autonomous systems. This aligns closely with the broader paradigm of “algorithm aversion” or “robophobia,” wherein individuals manifest systemic distrust toward automated decisions in high-stakes environments like healthcare, even when algorithms statistically outperform human experts [14]. Our empirical findings substantiate that patients fear GAI deployment may lead to a loss of clinician empathy, the systemic reinforcement of healthcare inequalities, and a breakdown in therapeutic rapport, transforming a deeply human interaction into a mechanical, potentially biased transaction.

The second and fourth dimensions, *Security Anxiety* and *Accuracy Anxiety*, delineate tangible algorithmic risks unique to generative architectures. Unlike traditional analytical AI, GAI is prone to “hallucinations”—the generation of plausible-sounding but clinically incorrect or completely fabricated medical information [38]. The *Security Anxiety* factor underscores the intensifying public discourse surrounding data privacy, electronic health record (EHR) vulnerabilities, and the unauthorized commercialization of sensitive medical histories [2]. Crucially, the emergence of *Accuracy Anxiety* as a distinct factor highlights a psychological phenomenon where GAI tools present health information with an absolute, authoritative tone. This lack of cognitive flexibility or nuance in AI outputs can exacerbate health-related obsessions, fuel health anxieties, and trigger behavioral disorders such as cyberchondria, as patients struggle to contextualize the “absolute” conclusions delivered by the machine.

From a psychometric perspective, the AI-HAS demonstrated exemplary internal consistency regarding reliability; both Cronbach’s alpha (0.918) and McDonald’s omega (0.916) coefficients indicated high inter-item relatedness and measurement precision [31]. However, a notable finding emerged regarding the scale’s stability over time: the test–retest reliability coefficient indicated an overall scale stability of 0.409. Rather than pointing to a structural flaw in the items, this statistical result highlights a fundamental theoretical characteristic of anxiety regarding artificial intelligence (AI). Anxiety toward AI in healthcare settings functions predominantly as a dynamic “state” rather than a stable “personality trait” independent of specific circumstances. Public perception of artificial intelligence is currently volatile; it is heavily influenced by factors such as daily media reports, fluctuating institutional trust, and the immediate severity of the patient’s medical condition at the time of assessment. Furthermore, situational variations may have arisen due to the limited sample size at the retest stage (*n* = 26), the shift to remote data collection, and the potential for participant fatigue resulting from the multi-dataset structure of the broader research project. This situation can be interpreted as representing a temporary methodological fluctuation rather than a threat to the scale’s fundamental validity.

Regarding the validity of the AI-HAS, Confirmatory Factor Analysis (CFA) fit indices (CMIN/DF = 2.510, CFI = 0.930, RMSEA = 0.082) confirmed that the structure derived from Exploratory Factor Analysis adequately reflects the empirical data structure of the four-factor model and meets standard structural equation modeling threshold values [25]. An examination of the explained variance ratios of the factors also provides significant evidence supporting the validity of the established structure. Furthermore, the scale’s discriminative ability and its capacity to serve its intended purpose have yielded important evidence regarding its construct validity.

### 4.1. Practical and Clinical Implications

The four-dimensional AI-HAS offers a preliminary framework for describing adult outpatients’ concerns about healthcare AI. In research and service-planning projects, item and subscale responses may help identify topics for patient information materials and staff communication training. Aggregated findings could guide local needs assessments, provided that the scale’s performance is evaluated in the intended population.

In clinical encounters, the item content may help structure conversations about AI-supported care. Concerns about negative outcomes could prompt discussion of individualized care and the clinician’s role. Security concerns could guide explanations of data protection and safeguards against erroneous decisions. Integration concerns could highlight the need to maintain clinician–patient communication. In addition, accuracy concerns could inform explanations of uncertainty and the limitations of AI-provided health information. These proposed applications are consistent with patient research emphasizing safety and clinician oversight and with WHO guidance on transparency, autonomy and privacy [5,6].

These applications require further evaluation. The present study did not establish clinical cut-offs, test whether scale-guided communication improves patient outcomes, or assess responsiveness to intervention. The relatively low test–retest correlations also limit interpretation of individual score changes. Accordingly, the AI-HAS currently provides preliminary evidence for research use. Routine clinical screening and treatment decisions would require additional validation. Future studies should assess feasibility and examine whether interventions designed around the identified concerns improve patient understanding and anxiety outcomes.

### 4.2. Limitations and Future Directions

Several limitations must be acknowledged. First, data collection relied on convenience sampling at a single tertiary academic medical center (Kırıkkale University Faculty of Medicine Hospital in Türkiye), which limits the immediate generalizability of the findings to broader socio-demographically diverse national populations. Second, the initial linguistic and face validity pilot phase was conducted with a relatively homogeneous group of health science students, which may not fully capture the comprehension barriers experienced by elderly or less-educated outpatient demographics. Lastly, this study focused strictly on structural and internal reliability; hence, criterion-related validity (e.g., correlating AI-HAS scores with established generalized anxiety measures like the GAD-7) was not empirically assessed. Finally, the test–retest reliability coefficients of the scale are relatively low. However, considering that the correlations between administrations are significant and the internal consistency coefficients are quite high, it can be concluded that the scale is reliable.

Future research should prioritize multi-center, cross-cultural validations of the AI-HAS to establish its invariant properties across diverse demographic strata. Investigators are encouraged to incorporate convergent and discriminant validity metrics to delineate how GAI-specific anxiety diverges from generalized or technology-related anxieties. Pragmatically, the AI-HAS can serve as a vital screening tool for healthcare administrators and policymakers to identify patient anxiety thresholds, enabling the design of targeted educational interventions and transparent AI-integration policies that foster patient trust and mitigate systemic resistance.

## 5. Conclusions

This study developed the AI-HAS as an 18-item measure of adult outpatients’ anxiety about AI use in healthcare. The scale comprises four dimensions: Anxiety Regarding Negative Outcomes, Security Anxiety, AI Integration Anxiety, and Accuracy Anxiety. Confirmatory analysis provided preliminary support for this structure, and the scale showed high internal consistency (Cronbach’s α = 0.918; McDonald’s ω = 0.916). The test–retest correlation was 0.409 in 26 participants, indicating that temporal stability requires further evaluation.

For healthcare researchers, the main takeaway is that the AI-HAS provides an initial tool for examining distinct dimensions of patients’ anxiety about AI-supported care. This can inform research into patient communication and educational needs. Further validation in larger, more diverse clinical samples is needed, including additional assessment of temporal stability and relationships with established anxiety measures.

## Figures and Tables

**Table 1:** Demographic Characteristics of the EFA, CFA, and Stability Samples.

Demographic Characteristic	EFA Sample (*n* = 369)	CFA Sample (*n* = 225)	Stability Sample (*n* = 26)
**Female**	186 (50.4%)	112 (49.7%)	16 (61.5%)
**Male**	183 (49.6%)	113 (50.3%)	10 (38.5%)
**City Center**	290 (78.6%)	181 (80.4%)	20 (76.9%)
**Rural**	79 (21.4%)	44 (19.6%)	6 (23.1%)
**Educational Attainment**			
Primary Education	44 (11.9%)	18 (8.0%)	3 (11.5%)
High School	100 (27.1%)	79 (35.1%)	8 (30.8%)
Associate degree	47 (12.7%)	31 (13.8%)	1 (3.8%)
Bachelor’s Degree	156 (42.3%)	87 (38.7%)	11 (42.3%)
Graduate Degree	22 (6.0%)	10 (4.4%)	3 (11.5%)
**Age Distribution**			
18–19 years	27 (7.3%)	5 (2.2%)	0 (0%)
20–30 years	154 (41.7%)	91 (40.4%)	15 (57.7%)
31–40 years	65 (17.6%)	46 (20.4%)	1 (3.8%)
41–50 years	80 (21.7%)	43 (19.1%)	3 (11.5%)
Over 50 years	43 (11.7%)	40 (17.8%)	7 (26.9%)

**Table 2:** Factor Correlation Matrix.

Factor	1	2	3	4
**1**	1.00	0.292	0.287	0.315
**2**	0.292	1.00	0.309	0.339
**3**	0.287	0.309	1.00	0.264
**4**	0.315	0.339	0.264	1.00

*Extraction Method: Principal Axis Factoring. Rotation Method: Oblimin with Kaiser Normalization*.

**Table 3:** Results of the Factor Analysis of the Scale by Factors.

Factor	No	Items	Communalities	F1	F2	F3	F4
F1: Anxiety Regarding Negative Outcomes	I23	The use of artificial intelligence in healthcare concerns me.	0.716	0.749			
	I22	The thought that artificial intelligence will replace healthcare professionals in the near future concerns me.	0.779	0.747			
	I16	I am concerned that artificial intelligence will disrupt the healthcare system in the future.	0.715	0.705			
	I20	I am concerned that artificial intelligence will make me a hypochondriac.	0.688	0.651			
	I25	Artificial intelligence may discriminate among people.	0.699	0.563			
	I24	My specific health condition may not be adequately considered by artificial intelligence.	0.726	0.539			
	I12	I am concerned that artificial intelligence will replace humans.	0.708	0.506			
F2: Security Anxiety	I2	I am concerned that I might become a victim of fraud when using artificial intelligence applications in healthcare.	0.724		0.812		
	I1	I am concerned that my information will be stolen when using artificial intelligence applications in healthcare.	0.549		0.794		
	I3	I am concerned about whether artificial intelligence systems will make accurate decisions regarding my illness.	0.628		0.708		
	I4	I am concerned that an artificial intelligence-supported system may make an incorrect diagnosis.	0.779		0.684		
F3: AI Integration Anxiety	I7	I am concerned that the use of artificial intelligence will weaken doctor-patient communication.	0.752			0.786	
	I8	The reduction in the human touch in healthcare services concerns me.	0.649			0.776	
	I6	The idea of decisions regarding my treatment being made by a machine concerns me.	0.561			0.690	
	I5	The involvement of artificial intelligence in healthcare decisions concerns me.	0.632			0.607	
F4: Accuracy Anxiety	I14	Artificial intelligence tools making definitive diagnostic recommendations like a doctor concerns me.	0.645				0.770
	I15	Artificial intelligence tools presenting health-related information as if it is absolute certainty concerns me.	0.465				0.761
	I13	Artificial intelligence can cause excessive behavioral disorders and anxiety in individuals with health obsessions.	0.427				0.755
	**Eigen Values**			3.38	3.13	2.71	2.62
	**Explanation Variance Rates**			18.80	17.37	15.05	14.58
	**Total Explanation Variance Rate**			65.80			

Bold text are used for emphasis.

**Table 4:** Confirmatory Factor Analysis Fit Index Values.

Indexes	Values	Acceptable Fit Values	Interpretation
X^2^ (df, *p*)	328.4 (128), *p* < 0.001)		Significant
RMSEA	0.082 [0.074, 0.097]	RMSEA < 0.08	Acceptable Fit
CFI	0.930	CFI > 0.90	Acceptable Fit
IFI	0.931	IFI > 0.90	Acceptable Fit
TLI	0.916	TLI > 0.90	Acceptable Fit
SRMR	0.056	SRMR < 0.08	Acceptable Fit

**Table 5:** Standardized regression weights.

F1: Anxiety Regarding Negative Outcomes		F2: Security Anxiety		F3: AI Integration Anxiety		F4: Accuracy Anxiety	
I	Estimate	I	Estimate	I	Estimate	I	Estimate
I23	0.746	I2	0.662	I5	0.895	I13	0.713
I22	0.628	I1	0.779	I6	0.910	I15	0.891
I16	0.758	I3	0.807	I8	0.842	I14	0.874
I20	0.691	I4	0.827	I7	0.776		
I25	0.572						
I24	0.604						
I12	0.684						

**Table 6:** Convergent Validity and Composite Reliability Results of the Scale Dimensions.

Factors	CR	Interpretation CR > 0.70	AVE	Interpretation AVE > 0.50
**F1: Anxiety Regarding Negative Outcomes**	0.852	Perfect	0.450	Acceptable
**F2: Security Anxiety**	0.854	Perfect	0.596	Perfect
**F3: AI Integration Anxiety**	0.916	Perfect	0.733	Perfect
**F4: Accuracy Anxiety**	0.868	Perfect	0.689	Perfect

**Table 7:** Item-Factor Correlations.

F1 Anxiety Regarding Negative Outcomes		F2 Security Anxiety		F3 AI Integration Anxiety		F4 Accuracy Anxiety	
I	r	I	r	I	r	I	r
I23	0.785	I2	0.870	I7	0.812	I14	0.900
I22	0.778	I1	0.850	I8	0.805	I15	0.886
I16	0.782	I3	0.845	I6	0.870	I13	0.819
I20	0.722	I4	0.811	I5	0.823		
I25	0.561						
I24	0.659						
I12	0.701						

Note. *n* = 369; *p* < 0.001.

**Table 8:** Item Discrimination Power.

F1 Anxiety Regarding Negative Outcomes		F2 Security Anxiety		F3 AI Integration Anxiety		F4 Accuracy Anxiety	
I	t	I	t	I	t	I	t
I23	14.61	I2	13.61	I7	12.68	I14	14.56
I22	14.74	I1	13.23	I8	13.80	I15	13.47
I16	15.46	I3	12.51	I6	16.74	I13	10.65
I20	13.87	I4	11.22	I5	14.46		
I25	7.59						
I24	11.28						
I12	15.92	**F1** **: 22.70**	**F2: 16.02**	**F3: 20.02**	**F4: 15.75**	**Total: 30.47**	

Note. *n* = 100; *df*: 198; *p* < 0.001. Bold values are used for emphasis.

**Table 9:** Internal Consistency and Stability Coefficients.

Factors	Number of Items	Omega	Cronbach’s Alpha	Stability *
F1: Anxiety Regarding Negative Outcomes	7	0.837	0.836	0.395
F2: Security Anxiety	4	0.853	0.864	0.402
F3: AI Integration Anxiety	4	0.843	0.847	0.458
F4: Accuracy Anxiety	3	0.846	0.837	0.389
**Total**	**18**	**0.916**	**0.918**	**0.409**

Note. * *n* = 26; *p* < 0.001.

## Data Availability

The data presented in this study are available from the corresponding author upon reasonable request. The dataset forms part of an ongoing research project and is intended to be used in a subsequent structural equation modeling study. Therefore, it has not yet been deposited in a public repository. The dataset will be made publicly available after the completion of the related research process.
